# Clinical outcomes and repair integrity of arthroscopic rotator cuff repair using suture-bridge technique with or without medial tying: prospective comparative study

**DOI:** 10.1186/s13018-018-0921-z

**Published:** 2018-08-28

**Authors:** Kyung Cheon Kim, Hyun Dae Shin, Woo-Yong Lee, Kyu-Woong Yeon, Sun-Cheol Han

**Affiliations:** 1 Shoulder Center, Department of Orthopedic Surgery, TanTan Hospital, Daejeon, South Korea; 2 Department of Orthopedic Surgery, Regional Rheumatoid and Degenerative Arthritis Center, Chungnam National University Hospital, Chungnam National University School of Medicine, 266 Munwha-ro, Jung-gu, Daejeon, 35015 South Korea

**Keywords:** Rotator cuff, Suture-bridge, Knotless, Medial knot tying

## Abstract

**Background:**

There have been few studies comparing clinical and radiological outcomes between the conventional and knotless suture-bridge techniques. The purpose of this study was to evaluate and compare the functional outcomes and repair integrity of arthroscopic conventional and knotless suture-bridge technique for full-thickness rotator cuff tears.

**Methods:**

We prospectively followed 100 consecutive patients (100 shoulders) with full-thickness rotator cuff tears treated with the arthroscopic conventional or knotless suture-bridge technique from October 2012 to July 2014. Enrolled patients returned for follow-up functional evaluations at 1 and 2 years after the operation. There were four outcome measures in this study: American Shoulder and Elbow Surgeons (ASES) scores, Shoulder Rating Scale of the University of California at Los Angeles (UCLA) scores, Constant scores, and visual analog scale (VAS) pain scores. Enrolled patients returned for follow-up magnetic resonance imaging or ultrasonography evaluation to confirm the integrity of the repaired cuff at 6 months post-operation (97% follow-up rate). Also, we investigated the preoperative cuff retraction of enrolled patients using preoperative MRI to find out correlation between the stage of cuff retraction and re-tear rate.

**Results:**

At final follow-up, the average UCLA, ASES, Constant, and VAS scores had improved significantly to 32.5, 88.0, 80.4, and 1.3, respectively, in the conventional suture-bridge technique group and to 33.0, 89.7, 81.2, and 1.2, respectively, in the knotless suture-bridge technique group. The UCLA, ASES, Constant, and VAS scores improved in both groups after surgery (all *p* < 0.001), and there were no significant differences between the two groups at 2-year follow-up (*p* = 0.292, 0.359, 0.709, and 0.636, respectively). The re-tear rate of repaired rotator cuffs was 16.3% (8/49 shoulders) in the conventional suture-bridge technique group and 29.2% (14/48 shoulders) in the knotless suture-bridge technique group; this difference was not significant (*p* = 0.131). There were no significant differences between the re-tear rate of the two groups in the Patte stage I and II (*p* = 0.358 and 0.616).

**Conclusions:**

The knotless suture-bridge technique showed comparable functional outcomes to those of conventional suture-bridge techniques in medium-to-large, full-thickness rotator cuff tears at short-term follow-up. The knotless suture-bridge technique had a higher re-tear rate compared with conventional suture-bridge technique, although the difference was not significant.

## Background

Rotator cuff tears (RCTs) comprise the majority of shoulder lesions in adult patients. The prevalence of RCTs among the general population is 22.1% and increases with age [1]. Despite widespread use, rotator cuff repair (RCR) surgeries do not always lead to clinically satisfactory outcomes; indeed, the failure rate of RCR is reportedly 40–50% [2–4]. Rotator cuff reattachment to the bone during RCR is a challenging clinical problem. To address this problem, surgical repair techniques have been continually developed over time in an attempt to reduce re-tear rates and improve functional outcomes. Recently, arthroscopic transosseous-equivalent suture-bridge RCR, namely, the suture-bridge technique (SBT), has been widely used to enhance healing at the site of tendon insertion of the repaired rotator cuff. This repair method involves insertion of a medial row with suture anchors that utilize mattress repairs [5–8]. However, techniques that employ a knotted medial row of anchors have been suspected to compromise vascular inflow to the healing tendon and increase the risk of type II failure (i.e., medial row failure), which is very difficult to treat [9–13]. More recently, knotless RCR techniques that involve application of knotless medial anchors, to improve vascular circulation and prevent type II failure, have been introduced [14–18].

Despite these innovations and documented benefits in a laboratory setting, postoperative clinical and radiological outcomes of newer SBTs, at short- to medium-term follow-up, have been equivocal [14, 18–20]. Moreover, few studies comparing clinical and radiological outcomes between the conventional and knotless SBT have been reported.

The purpose of this study was to evaluate and compare functional outcomes and repair integrity between arthroscopic conventional and knotless SBT for full-thickness RCTs.

## Methods

### Patient selection

We prospectively followed 100 consecutive patients (100 shoulders) with full-thickness RCTs treated with arthroscopic conventional or knotless SBT from October 2012 to July 2014 at our institute. A conventional SBT was used in the first 50 consecutive shoulders, and a knotless SBT was used in the next 50 consecutive shoulders (Table 1). We included full-thickness supraspinatus or infraspinatus tears 1–4 cm in length in the anterior-to-posterior dimension. We excluded patients with the following: (1) full-thickness RCTs smaller than 1 cm or larger than 4 cm in the anterior-to-posterior dimension, (2) a full-thickness subscapularis tear requiring concomitant repair, (3) neurological involvement, (4) revision operation, (5) operation after conversion of an advanced partial-thickness RCT to a full-thickness lesion, and (6) advanced arthritic changes in the glenohumeral joint. The demographic characteristics of the conventional and knotless SBT groups are listed in Table 1.

**Table 1:** Demographic and surgical data of the two study groups

Variable	Conventional SBT	Knotless SBT	*p* value*
Number of patients	50	50	
Age at surgery, years	59.40 ± 7.45 (41–76)	59.90 ± 7.66 (47–74)	0.741
Gender, male	28 (56.0%)	24 (48.0%)	0.423
Affected shoulder, right	29 (58.0%)	34 (68.0%)	0.841
Duration of symptoms, months	5.86 ± 6.00 (1–36)	6.10 ± 9.05 (1–48)	0.876
Current smoker	9 (18.0%)	10 (20.0%)	0.532

Data are expressed as mean ± standard deviation (range) or number (percentage)

*SBT* Suture-bridge technique

*Paired *t* test; *p* < 0.05 denotes statistical significance

### Surgical technique

**Fig. 1:**
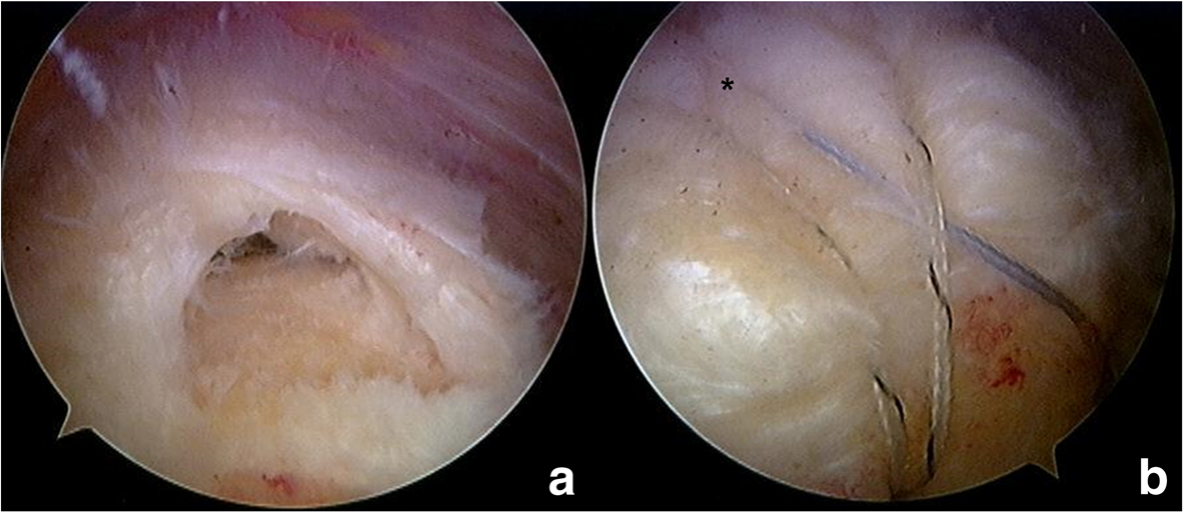
**a** Arthroscopic view showing a rotator cuff tear involving the supraspinatus. **b** The arthroscopic view from the lateral portal shows complete repair of an rotator cuff tear using the knotless suture-bridge technique without medial tying (*)

All operations were performed by a single surgeon (first author) in the beach-chair position with the patient under general anesthesia. During conventional SBT, we used one or two Bio-Corkscrew suture anchors (4.5 or 5.5 mm according to the tear size; Arthrex, Naples, FL, USA), containing a suture eyelet and loaded with two No. 2 non-absorbable braided sutures, placed just lateral to the articular surface of the humeral head. The sutures perforated the tendon in a horizontal mattress stitch configuration, with an identical procedure then applied for the second medial anchor [5]. To establish the lateral row, suture bridge repair was achieved with two or three 4.5-mm knotless anchors (Bio-PushLock; Arthrex) that were fully inserted perpendicular to the cortical surface of the humerus, distal to the footprint anchor in conjunction with one suture from each medial anchor (Fig. 1).

**Fig. 2:**
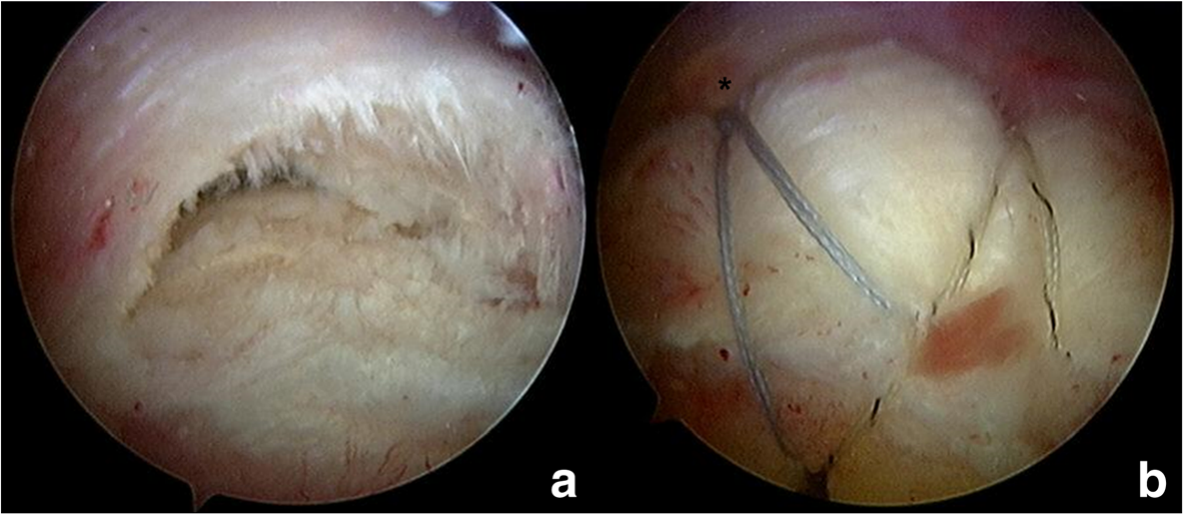
**a** Arthroscopic view showing a rotator cuff tear involving the supraspinatus. **b** The arthroscopic view from the lateral portal shows complete repair of a rotator cuff tear using the conventional suture-bridge technique with medial tying (*)

For the knotless SBT, one or two of the same suture anchors were placed just lateral to the articular surface of the humeral head. In shoulders with a suture anchor inserted into the medial row, four suture limbs in the suture anchor were passed through the reduced tendon in an alternative configuration; a pilot hole for the 4.5-mm knotless anchors (Bio-PushLock; Arthrex) was prepared approximately 5–10 mm distal to the lateral edge of the greater tuberosity. Without tying the medial row, the same two suture limbs were linked to the knotless anchor, which was placed within the pilot hole. These steps were then repeated for a second knotless anchor. In shoulders with two suture anchors inserted into the medial row, four limbs from the sutures in the first suture anchor were passed through the tendon in an alternative configuration. Then, the second suture anchor was inserted and one suture was removed. After tying the remaining suture twice to prevent sliding, two suture limbs were passed through the tendon in a mattress configuration. Without tying the medial row, the same two limbs, and one limb of the second anchor, were linked to the knotless anchor, which was placed within the pilot hole. These steps were again repeated for a second knotless anchor (Fig. 2).

The maximum anterior-to-posterior length of the RCTs was measured using a calibrated probe, introduced through the anterior or posterior portal while viewing the posterolateral or lateral portal under arthroscopic observation. The maximum medial-to-lateral length of the RCTs in oblique coronal images on preoperative T2-weighted magnetic resonance imaging (MRI) was estimated; because of the considerable variation in the medial-to-lateral length according to the position of the shoulder, we measured the tears on MRI instead of arthroscopy (Table 2). All of the measurements were performed by the first author. Important clinical differences between the conventional and knotless SBT groups are listed in Table 2.

**Table 2:** Clinical and surgical data of the two study groups

Variable	Conventional SBT	Knotless SBT	*p* value*
Total number of patients	50	50	
Clinical evaluation			
1-year follow-up	47 (94.0%)	48 (96.0%)	0.646
2-year follow-up	49 (98.0%)	47 (94.0%)	0.307
Postoperative radiologic evaluation	49 (98.0%)	48 (96.0%)	0.941
MRI	32	31	
US	17	17	
Tear size, mm			
Anterior-to-posterior	2.51 (1.6–4.0)	2.53 (1.5–3.9)	0.918
Medial-to-lateral	1.96 (0.8–3.5)	1.97 (0.5–3.5)	0.906
Cuff retraction (Patte stage)			0.188
Stage I	31 (63.3%)	23 (47.9%)	
Stage II	18 (36.7%)	25 (52.1%)	
Stage III	0	0	
Subacromial decompression	47 (94.0%)	47 (94.0%)	1.000
Biceps tenotomy	25 (50.0%)	25 (50.0%)	1.000

Data are expressed as mean (range) or number (percentage)

*SBT* suture-bridge technique, *MRI* magnetic resonance imaging, *US* ultrasonography

*Paired *t* test and *χ*^2^ test; *p* < 0.05 denotes statistical significance

### Postoperative management

All patients received standardized pre- and perioperative care at a single hospital. The same treatment regimen was prescribed to all patients, regardless of the repair status of the articular-side rotator cuff. Postoperatively, we recommended the use of a shoulder-immobilizing sling with an abduction pillow and provided instructions to maintain the shoulder at 30–40° internal rotation and 20° abduction. The patients performed gentle passive forward flexion exercises of the affected arm during the second postoperative week. The sling and abduction pillow were removed at 6 weeks postoperatively, and active mobilization was started. Active resistance-based muscle-strengthening exercises were started at 12 weeks postoperatively using Thera-Band equipment (HCM-Hygenic Corp., Batu Gajah, Malaysia). At 3–4 months after surgery, the patients were permitted to perform light activities, with sports participation and heavy labor being allowed after 6 months.

### Clinical and radiological evaluation

Enrolled patients returned for a follow-up functional evaluation at 1 and 2 years after the operation. Clinical data were collected preoperatively and postoperatively at the 1- and 2-year follow-ups by two orthopedic surgeons. There were four outcome measures in this study: American Shoulder and Elbow Surgeons (ASES) scores, Shoulder Rating Scale of the University of California at Los Angeles (UCLA) scores, Constant scores, and visual analog scale (VAS) pain scores. Active range of motion was measured by goniometry, while passive range of motion was not measured. The range of motion was measured with the patient in a standing position, and external rotation was assessed while the patient was standing with the arm in an adducted position.

Enrolled patients returned for follow-up MRI or ultrasonography (US) evaluation to confirm the integrity of the repaired cuff at 6 months post-operation (97% follow-up rate). One specialized musculoskeletal radiologist performed all follow-up US examinations using an IU-22 system (Philips Healthcare, Bothell, WA, USA). The MRI or US images were evaluated by an experienced radiologist. A recurrent tendon defect was diagnosed by US when a distinct hypoechoic or mixed hyper- and hypoechoic defect was visualized in both the transverse and longitudinal planes. A full-thickness re-tear was diagnosed when a focal defect was present in the rotator cuff, into which the deltoid muscle could be compressed with a probe to separate the torn tendon ends, or when the cuff retracted to such an extent that the torn ends could be distinctly visualized. MRI was used to classify the integrity of the tendon into one of two categories: (1) intact (sufficient thickness, Sugaya types I and II) or (2) insufficient/unhealed/re-torn [ranging from insufficient thickness (< 50% normal cuff thickness) to discontinuity, Sugaya types III–V] [21].

Also, we investigated the preoperative cuff retraction of enrolled patients returned for follow-up MRI or US evaluation using preoperative MRI to find out correlation between the stage of cuff retraction and re-tear rate. The degree of cuff retraction in the coronal plane of preoperative MRI was assessed by Patte classification: (1) stage I is a tear with minimal retraction, (2) stage II is a tear retracted medial to the humeral head footprint but not to the glenoid, and (3) stage III is a tear retracted to the level of the glenoid [22]. Important clinical differences between the groups are listed in Table 2.

### Statistical analyses

According to a two-sided significance level of 0.05 and a power of 80%, we prospectively enrolled patients in this study. The sample size was calculated with consideration of each outcome measure, namely, the UCLA, ASES, Constant, and VAS scores. The sample size required to achieve an 80% power was 8, 10, 12, and 13 for the UCLA, ASES, Constant scores, and VAS, respectively. A minimum of 20 patients, which is the maximal sample size among the outcomes, was required to satisfy the conditions (the power of 80% and 20% maximum follow-up loss of patients). For the statistical analysis, the paired *t* test and *χ*^2^ test were used to assess pre- and postoperative differences between the groups. The SPSS software package was used for all statistical analyses (ver. 12.0; SPSS Inc., Chicago, IL, USA) with the *α* level set at 0.05.

## Results

Preoperatively, no significant differences were observed between the groups in the mean UCLA, ASES, Constant, or VAS scores (*p* = 0.175, 0.111, 0.432, and 0.890, respectively; Table 3). At 1-year follow-up, the average UCLA, ASES, Constant, and VAS scores had improved significantly to 31.1, 83.5, 72.1, and 1.9, respectively, in the conventional SBT group and to 31.7, 84.4, 73.9, and 1.4, respectively, in the knotless SBT group (Table 4). The UCLA, ASES, Constant, and VAS scores improved in both groups after surgery (all *p* < 0.001); however, there was no significant difference between the two groups at 1-year follow-up (*p* = 0.434, 0.733, 0.437, and 0.951, respectively; Table 5).

**Table 3:** Comparison of preoperative scores between the two study groups

Variable	Conventional SBT (50)	Knotless SBT (50)	*p* value*
UCLA	19.96 ± 4.30	18.46 ± 4.92	0.175
ASES	53.89 ± 15.15	51.43 ± 15.84	0.111
Constant	57.50 ± 13.15	57.98 ± 20.24	0.432
VAS	5.28 ± 1.88	5.79 ± 1.82	0.890

Numbers in parentheses are the numbers of patients in each group

Data are expressed as mean ± standard deviation

*SBT* suture-bridge technique, *UCLA* Shoulder Rating Scale of the University of California at Los Angeles, *ASES* American Shoulder and Elbow Surgeons score, *VAS* visual analog scale pain score

*Paired *t* test; *p* < 0.05 denotes statistical significance

**Table 4:** Comparison between the preoperative findings and postoperative clinical outcomes at 1-year follow-up

	Preoperative	1-year follow-up	*p* value*
UCLA			
Conventional SBT (47)	19.96 ± 4.41	31.09 ± 4.23	0.000
Knotless SBT (48)	18.46 ± 4.92	31.67 ± 2.83	0.000
ASES			
Conventional SBT (47)	52.95 ± 15.12	83.54 ± 15.26	0.000
Knotless SBT (48)	51.43 ± 15.84	84.42 ± 8.69	0.000
Constant			
Conventional SBT (47)	57.51 ± 13.51	72.06 ± 11.46	0.000
Knotless SBT (48)	57.98 ± 20.24	73.85 ± 10.87	0.000
VAS			
Conventional SBT (47)	5.30 ± 1.86	1.91 ± 2.05	0.000
Knotless SBT (48)	5.79 ± 1.82	1.94 ± 1.44	0.000

Numbers in parentheses are the numbers of patients in each group

Data are expressed as mean ± standard deviation

*SBT* suture-bridge technique, *UCLA* Shoulder Rating Scale of the University of California at Los Angeles, *ASES* American Shoulder and Elbow Surgeons score, *VAS* visual analog scale pain score

*Paired *t* test; *p* < 0.05 denotes statistical significance

**Table 5:** Comparison of clinical outcomes between the two study groups at 1-year follow-up

	Conventional SBT (47)	Knotless SBT (48)	*p* value*
UCLA	31.09 ± 4.23	31.67 ± 2.83	0.434
ASES	83.54 ± 15.26	84.42 ± 8.69	0.733
Constant	72.06 ± 11.46	73.85 ± 10.87	0.437
VAS	1.91 ± 2.05	1.94 ± 1.44	0.951

Numbers in parentheses are the numbers of patients in each group

Data are expressed as mean ± standard deviation

*SBT* suture-bridge technique, *UCLA* Shoulder Rating Scale of the University of California at Los Angeles, *ASES* American Shoulder and Elbow Surgeons score, *VAS* visual analog scale pain score

*Paired *t* test; *p* < 0.05 denotes statistical significance

At final follow-up, the average UCLA, ASES, Constant, and VAS scores had improved significantly to 32.5, 88.0, 80.4, and 1.3, respectively, in the conventional SBT group and to 33.0, 89.7, 81.2, and 1.2, respectively, in the knotless SBT group (Table 6). The UCLA, ASES, Constant, and VAS scores improved in both groups after surgery (all *p* < 0.001), and there were no significant differences between the two groups at 2-year follow-up (*p* = 0.292, 0.359, 0.709, and 0.636, respectively; Table 7).

**Table 6:** Comparison between the preoperative findings and postoperative clinical outcomes at 2-year follow-up

	Preoperative	2-year follow-up	*p* value*
UCLA			
Conventional SBT (49)	19.94 ± 4.34	32.51 ± 2.72	0.000
Knotless SBT (47)	18.38 ± 4.95	32.98 ± 1.42	0.000
ASES			
Conventional SBT (49)	53.67 ± 15.22	87.97 ± 10.68	0.000
Knotless SBT (47)	51.35 ± 16.00	89.70 ± 7.53	0.000
Constant			
Conventional SBT (49)	57.71 ± 13.20	80.37 ± 12.85	0.000
Knotless SBT (47)	57.91 ± 20.46	81.17 ± 7.61	0.000
VAS			
Conventional SBT (49)	5.29 ± 1.90	1.27 ± 1.22	0.000
Knotless SBT (47)	5.79 ± 1.84	1.15 ± 1.18	0.000

Numbers in parentheses are the numbers of patients in each group

Data are expressed as mean ± standard deviation

*SBT* suture-bridge technique, *UCLA* Shoulder Rating Scale of the University of California at Los Angeles, *ASES* American Shoulder and Elbow Surgeons score, *VAS* visual analog scale pain score

*Paired *t* test; *p* < 0.05 denotes statistical significance

**Table 7:** Comparison of the clinical outcomes between the two groups at 2-year follow-up

	Conventional SBT (49)	Knotless SBT (47)	*p* value*
UCLA	32.51 ± 2.72	32.98 ± 1.42	0.292
ASES	87.97 ± 10.68	89.70 ± 7.53	0.359
Constant	80.37 ± 12.85	81.17 ± 7.61	0.709
VAS	1.27 ± 1.22	1.15 ± 1.18	0.636

Numbers in parentheses are the numbers of patients in each group

Data are expressed as mean ± standard deviation

*SBT* suture-bridge technique, *UCLA* Shoulder Rating Scale of the University of California at Los Angeles, *ASES* American Shoulder and Elbow Surgeons score, *VAS* visual analog scale pain score

*Paired *t* test; *p* < 0.05 denotes statistical significance

**Fig. 3:**
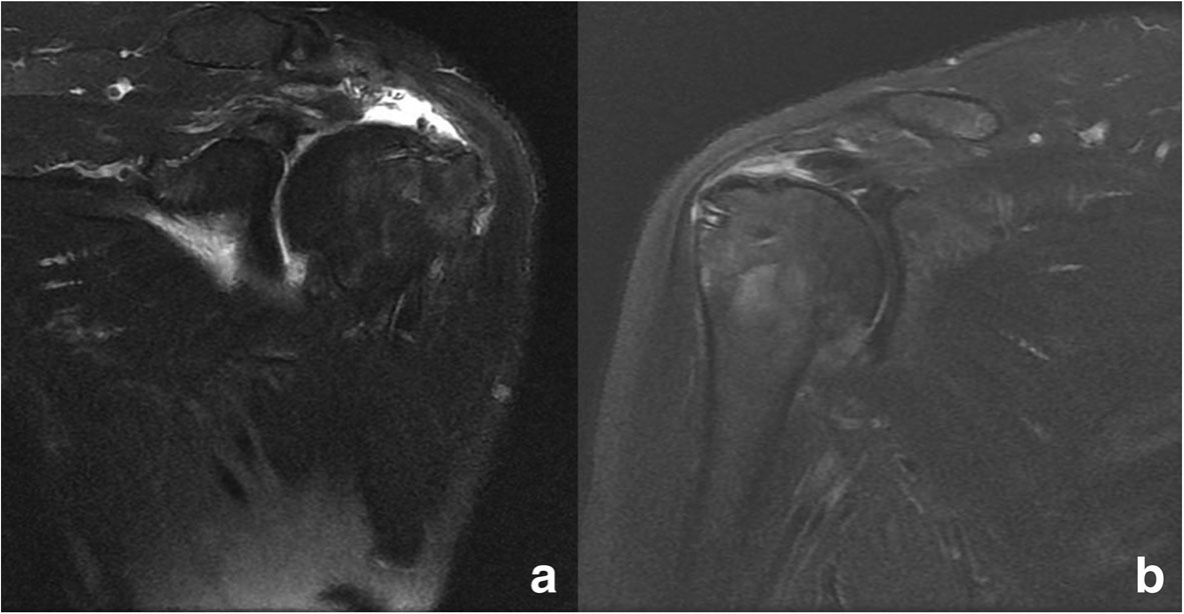
Follow-up T2-weighted sagittal magnetic resonance imaging at 6 months post-operation shows an unhealed tendon of a repaired rotator cuff (type I re-tear). **a** Conventional suture-bridge technique. **b** Knotless suture-bridge technique

**Fig. 4:**
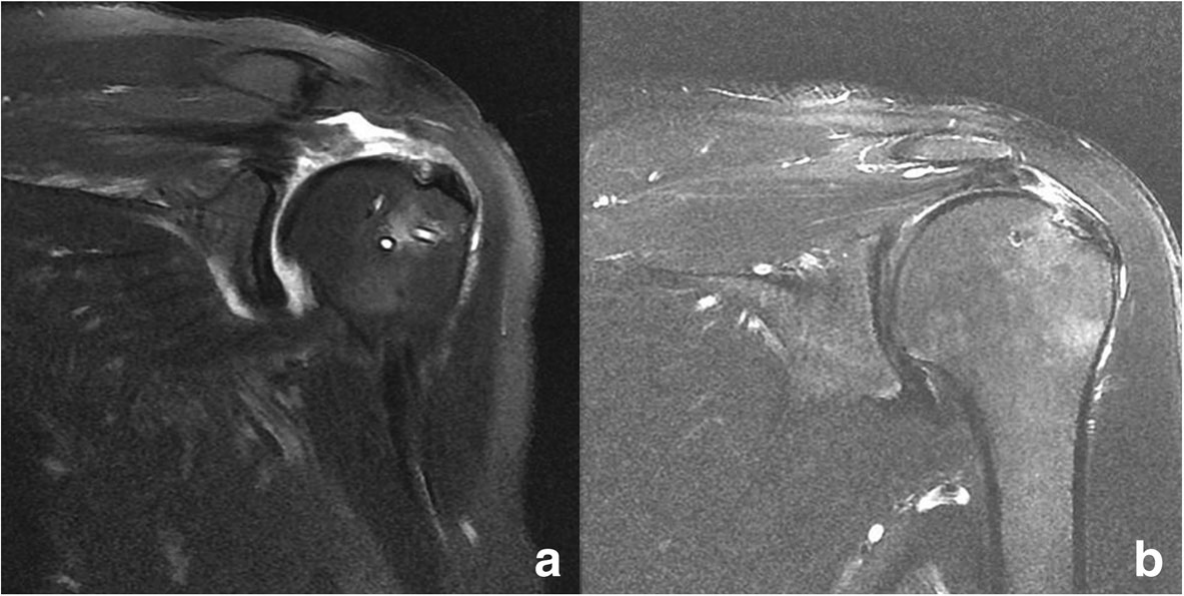
Follow-up T2-weighted magnetic resonance imaging at 6 months post-operation shows medially ruptured tendons and a healed footprint of a repaired rotator cuff (type II re-tear). **a** Conventional suture-bridge technique. **b** Knotless suture-bridge technique

The re-tear rate of the repaired rotator cuffs was 16.3% (8/49 shoulders) in the conventional SBT group and 29.2% (14/48 shoulders) in the knotless SBT group; this difference was not significant (*p* = 0.131). Two types of re-tear patterns were identified in both the conventional and knotless SBT groups: (1) unhealed tendons [2/8 (25%) and 6/14 (42.9%), respectively; Fig. 3] and (2) medially ruptured tendons with a healed footprint [6/8 (75%) and 8/14 (57.1%), respectively; Fig. 4]; different rate of re-tear pattern was statistically insignificant (*p* = 0.402). No intra- or perioperative complications were noted, and no patient showed neural injury, wound infection, or problems related to the suture anchor.

The re-tear rate was 22.2% (12/54 shoulders) in the Patte stage I and 23.3% (10/43 shoulders) in the stage II; this difference was not significant (*p* = 1.000). Also, the re-tear rate of the conventional SBT group was 16.1% (5/31 shoulders) in the stage I and 16.7% (3/18 shoulders) in the stage II; this difference was not significant (*p* = 1.000). The re-tear rate of the knotless SBT group was 30.4% (7/23 shoulders) in the stage I and 28.0% (7/25 shoulders) in the stage II; this difference was not significant (*p* = 1.000). And there were no significant differences between re-tear rate of the two groups in the Patte stage I and II (*p* = 0.358 and 0.616).

## Discussion

This study evaluated the clinical and radiographic results of knot-tying and knotless SBTs for RCTs. We applied an SBT without knot-tying to reduce tension overload at the suture-tendon interface of the medial row and the likelihood of medial cuff failure. We hypothesized that the biological advantages inherent to the knotless SBT would result in a higher healing rate than that associated with the conventional knot-tying SBT.

Suture-bridge RCR was introduced to improve the biomechanical outcomes of RCR [5–7]. It is more convenient to fasten the compromised tendon to the footprint anchor firmly using the suture limbs of the medial suture knots. With the SBT, the mean pressurized contact area between the tendon and the tuberosity insertion footprint has proven superior to that of the conventional double-row technique [6–8]. The SBT also shows better ultimate-to-load failure outcomes and less gap formation than the double-row technique [6–8]. This biomechanical superiority of the SBT may contribute, at least in part, to the low rate of structural failure of repaired cuffs. Moreover, double-row RCR, where each suture anchor is tied separately, is a technically demanding and time-consuming procedure [23].

In a study on medial rotator cuff failure after use of the arthroscopic double-row technique, Trantalis et al. [9] posited that the most likely causes of medial cuff failure were tension overload at the suture-tendon interface of the medial row, over-tensioning of the medial repair (resulting from an oblique and retrograde suture path), a relatively large hole in the rotator cuff caused by retrograde suture-passing instruments, and the effect of braided suture materials on their passage through the rotator cuff. In a study on medial rotator cuff failure after arthroscopic SBT, Cho et al. [12] suggested that the most likely causes of medial cuff failure were attempts to pass the tendon at the musculotendinous junction instead of at the tendon portion, which eventually renders the musculotendinous junction weak and vulnerable to re-tear, with an increased likelihood of strangulation, relatively rapid necrosis of the rotator cuff tendon at the medial row, and failure at the musculotendinous junction. To reduce the possibility of strangulation and relatively rapid necrosis of the rotator cuff tendon at the medial row, the type of knots used to secure the medial row, and the amount of tension used to tie them, should be considered carefully [14, 24].

Reducing unnecessary over-tension during RCT repair, developing new techniques that can distribute the load placed on the medial row may be important. Therefore, the knotless SBT may not only restore the footprint contact area of the rotator cuff, but may also reduce tension overload at the suture-tendon interface of the medial row [25, 26]. This may also reduce the likelihood of strangulation and relatively rapid necrosis of the rotator cuff tendon at the medial row [14]. As with arthroscopic double-row RCR, undue tension at the medial row due to use of conventional SBT may play a major role in repair failure [9]. This tension is usually concentrated at the medial row and is rarely exerted on the lateral row [9]. However, in knotless SBT, tension is usually concentrated at the lateral row and rarely at the medial row. Thus, pullout of the suture anchor at the lateral row after RCR may occur with the knotless SBT, particularly in patients with osteoporosis. Insufficient compression by the medial row suture limbs due to suture anchor pullout may compromise the healing of a repaired rotator cuff [27, 28].

The most frequently used portion of the Patte classification is retraction of the supraspinatus tendon in the coronal plane of MRI. The classification has been found to have moderate consent in assessing tear retraction in some reports [29, 30]. It has also been shown to have prognostic factor after RCR [31]. However, there were no significant differences of re-tear rate between Patte stage I and II. These results probably were due to confined tear size as inclusion criteria in this study, so that there was no RCT with Patte stage III. Also, there were no significant differences of re-tear rate between stages in each group. Therefore, there was no correlation between Patte stage and re-tear rate according to repair techniques.

This study had some limitations. First, although all of the US evaluations were performed by an experienced musculoskeletal radiologist, the technique remains examiner-dependent [32]. However, we did not perform the US examination ourselves to avoid surgeon bias [33]. Second, we could not evaluate preoperative muscle atrophy grades due to incomplete MRI data and the so-called Y-shaped view in some cases. Third, although there was no significant difference in the re-tear rate between groups, the likelihood of having a re-tear in the knotless SBT group was almost twice as high as in the conventional group. Therefore, this study might be under-powered to show this difference from the viewpoint of re-tear.

The study also had several strengths. First, the follow-up rate for the functional outcome and radiological evaluations was high (97%). Second, the study was prospective and enrolled homogeneous patients, with respect to tear size, who had full-thickness supraspinatus or infraspinatus tears 1–4 cm in length in the anterior-to-posterior dimension. Thus, the results can be considered reliable. Third, all of the operations were performed by the same surgeon.

## Conclusions

The knotless SBT showed comparable functional outcomes to those of conventional SBT in medium-to-large, full-thickness RCTs at short-term follow-up. The knotless SBT had a higher re-tear rate compared with conventional SBT, although the difference was not significant.

## Abbreviations

ASES: American Shoulder and Elbow Surgeons
MRI: Magnetic resonance imaging
RCR: Rotator cuff repair
RCT: Rotator cuff tear
SBT: Suture-bridge technique
UCLA: Shoulder Rating Scale of the University of California at Los Angeles
US: Ultrasonography
VAS: Visual analog scale

## References

[1] Minagawa H, Yamamoto N, Abe H, Fukuda M, Seki N, Kikuchi K (2013). Prevalence of symptomatic and asymptomatic rotator cuff tears in the general population: from mass-screening in one village. J Orthop.

[2] Elia F, Azoulay V, Lebon J, Faraud A, Bonnevialle N, Mansat P (2017). Clinical and anatomic results of surgical repair of chronic rotator cuff tears at ten-year minimum follow-up. Int Orthop.

[3] Heuberer PR, Smolen D, Pauzenberger L, Plachel F, Salem S, Laky B (2017). Longitudinal long-term magnetic resonance imaging and clinical follow-up after single-row arthroscopic rotator cuff repair: clinical superiority of structural tendon integrity. Am J Sports Med.

[4] Barnes LA, Kim HM, Caldwell JM, Buza J, Ahmad CS, Bigliani LU (2017). Satisfaction, function and repair integrity after arthroscopic versus mini-open rotator cuff repair. Bone Joint J.

[5] Park MC, ElAttrache NS, Ahmad CS, Tibone JE (2006). “Transosseous-equivalent” rotator cuff repair technique. Arthroscopy.

[6] Park MC, ElAttrache NS, Tibone JE, Ahmad CS, Jun BJ, Lee TQ (2007). Part I: footprint contact characteristics for a transosseous-equivalent rotator cuff repair technique compared with a double row repair technique. J Shoulder Elb Surg.

[7] Park MC, Tibone JE, ElAttrache NS, Ahmad CS, Jun BJ, Lee TQ (2007). Part II: biomechanical assessment for a footprintrestoring transosseous-equivalent rotator cuff repair technique compared with a double-row repair technique. J Shoulder Elb Surg.

[8] Quigley RJ, Gupta A, Oh JH, Chung KC, McGarry MH, Gupta R (2013). Biomechanical comparison of single-row, double-row, and transosseous-equivalent repair techniques after healing in an animal rotator cuff tear model. J Orthop Res.

[9] Trantalis JN, Boorman RS, Pletsch K, Lo IK (2008). Medial rotator cuff failure after arthroscopic double-row rotator cuff repair. Arthroscopy.

[10] Yamakado K, Katsuo S, Mizuno K, Arakawa H, Hayashi S (2010). Medial-row failure after arthroscopic double-row rotator cuff repair. Arthroscopy.

[11] Wang VM, Wang FC, McNickle AG, Friel NA, Yanke AB, Chubinskaya S (2010). Medial versus lateral supraspinatus tendon properties: implications for double-row rotator cuff repair. Am J Sports Med.

[12] Cho NS, Lee BG, Rhee YG (2011). Arthroscopic rotator cuff repair using a suture bridge technique: is the repair integrity actually maintained?. Am J Sports Med.

[13] Kim YK, Moon SH, Cho SH (2013). Treatment outcomes of single- versus double-row repair for larger than medium-sized rotator cuff tears: the effect of preoperative remnant tendon length. Am J Sports Med.

[14] Rhee YG, Cho NS, Parke CS (2012). Arthroscopic rotator cuff repair using modified Mason-Allen medial row stitch: knotless versus knot-tying suture bridge technique. Am J Sports Med.

[15] Vaishnav S, Millett PJ (2010). Arthroscopic rotator cuff repair: scientific rationale, surgical technique, and early clinical and functional results of a knotless self-reinforcing double-row rotator cuff repair system. J Shoulder Elb Surg.

[16] Barber FA, Drew OR (2012). A biomechanical comparison of tendon-bone interface motion and cyclic loading between single-row, triple-loaded cuff repairs and double-row, suture-tape cuff repairs using biocomposite anchors. Arthroscopy.

[17] Burkhart SS, Adams CR, Burkhart SS, Schoolfield JD (2009). A biomechanical comparison of 2 techniques of footprint reconstruction for rotator cuff repair: the SwiveLock-FiberChain construct versus standard double-row repair. Arthroscopy.

[18] Millett PJ, Espinoza C, Horan MP, Ho CP, Warth RJ, Dornan GJ (2017). Predictors of outcomes after arthroscopic transosseous equivalent rotator cuff repair in 155 cases: a propensity score weighted analysis of knotted and knotless self-reinforcing repair techniques at a minimum of 2 years. Arch Orthop Trauma Surg.

[19] Boyer P, Bouthors C, Delcourt T, Stewart O, Hamida F, Mylle G (2015). Arthroscopic double-row cuff repair with suture-bridging: a structural and functional comparison of two techniques. Knee Surg Sports Traumatol Arthrosc.

[20] Hug K, Gerhardt C, Hanevald H, Scheibel M (2015). Arthroscopic knotless-anchor rotator cuff repair: a clinical and radiological evaluation. Knee Surg Sports Traumatol Arthrosc.

[21] Sugaya H, Maeda K, Matsuki K, Moriishi J (2005). Functional and structural outcome after arthroscopic full-thickness rotator cuff repair: single-row versus dual-row fixation. Arthroscopy.

[22] Patte D (1990). Classification of rotator cuff lesions. Clin Orthop Relat Res.

[23] Kim KC, Rhee KJ, Shin HD, Kim YM (2007). A modified suture-bridge technique for a marginal dog-ear deformity caused during rotator cuff repair. Arthroscopy.

[24] Virk MS, Bruce B, Hussey KE, Thomas JM, Luthringer TA, Shewman EF (2017). Biomechanical performance of medial row suture placement relative to the musculotendinous junction in transosseous equivalent suture bridge double-row rotator cuff repair. Arthroscopy.

[25] Kim KC, Shin HD, Cha SM, Park JY (2014). Comparisons of retear patterns for 3 arthroscopic rotator cuff repair methods. Am J Sports Med.

[26] Ide J, Karasugi T, Okamoto N, Taniwaki T, Oka K, Mizuta H (2015). Functional and structural comparisons of the arthroscopic knotless double-row suture bridge and single-row repair for anterosuperior rotator cuff tears. J Shoulder Elb Surg.

[27] Kummer F, Hergan DJ, Thut DC, Pahk B, Jazrawi LM (2011). Suture loosening and its effect on tendon fixation in knotless double-row rotator cuff repairs. Arthroscopy.

[28] Leek BT, Robertson C, Mahar A, Pedowitz RA (2010). Comparison of mechanical stability in double-row rotator cuff repairs between a knotless transtendon construct versus the addition of medial knots. Arthroscopy.

[29] Kuhn JE, Dunn WR, Ma B, Wright RW, Jones G, Spencer EE (2007). Interobserver agreement in the classification of rotator cuff tears. Am J Sports Med.

[30] Spencer EE, Dunn WR, Wright RW, Wolf BR, Spindler KP, McCarty E (2008). Interobserver agreement in the classification of rotator cuff tears using magnetic resonance imaging. Am J Sports Med.

[31] Gladstone JN, Bishop JY, Lo IK, Flatow EL (2007). Fatty infiltration and atrophy of the rotator cuff do not improve after rotator cuff repair and correlate with poor functional outcome. Am J Sports Med.

[32] Park JY, Siti HT, Keum JS, Moon SG, Oh KS (2010). Does an arthroscopic suture bridge technique maintain repair integrity? A serial evaluation by ultrasonography. Clin Orthop Relat Res.

[33] Lafosse L, Brozska R, Toussaint B, Gobezie R (2007). The outcome and structural integrity of arthroscopic rotator cuff repair with use of the double-row suture anchor technique. J Bone Joint Surg Am.

